# Carcass and Primal Composition Predictions Using Camera Vision Systems (CVS) and Dual-Energy X-ray Absorptiometry (DXA) Technologies on Mature Cows

**DOI:** 10.3390/foods10051118

**Published:** 2021-05-18

**Authors:** José Segura, Jennifer L. Aalhus, Nuria Prieto, Ivy L. Larsen, Manuel Juárez, Óscar López-Campos

**Affiliations:** Lacombe Research and Development Centre, Agriculture and Agri-Food Canada, Lacombe, AB T4L 1W1, Canada; jose.seguraplaza@canada.ca (J.S.); jennifer.aalhus@outlook.com (J.L.A.); nuria.prietobenavides@canada.ca (N.P.); ivyleelarsen@gmail.com (I.L.L.); manuel.juarez@canada.ca (M.J.)

**Keywords:** beef primals, computer vision system, dual energy X-ray absorptiometry, mature cows, rib-eye camera, whole-side camera

## Abstract

This study determined the potential of computer vision systems, namely the whole-side carcass camera (HCC) compared to the rib-eye camera (CCC) and dual energy X-ray absorptiometry (DXA) technology to predict primal and carcass composition of cull cows. The predictability (R^2^) of the HCC was similar to the CCC for total fat, but higher for lean (24.0%) and bone (61.6%). Subcutaneous fat (SQ), body cavity fat, and retail cut yield (RCY) estimations showed a difference of 6.2% between both CVS. The total lean meat yield (LMY) estimate was 22.4% better for CCC than for HCC. The combination of HCC and CCC resulted in a similar prediction of total fat, SQ, and intermuscular fat, and improved predictions of total lean and bone compared to HCC/CCC. Furthermore, a 25.3% improvement was observed for LMY and RCY estimations. DXA predictions showed improvements in R^2^ values of 26.0% and 25.6% compared to the HCC alone or the HCC + CCC combined, respectively. These results suggest the feasibility of using HCC for predicting primal and carcass composition. This is an important finding for slaughter systems, such as those used for mature cattle in North America that do not routinely knife rib carcasses, which prevents the use of CCC.

## 1. Introduction

In Canada, ~425,000 mature cows are harvested annually, producing over ~100,000 Tm of meat [1]. Recently, the reduced availability of cattle and the increase in beef demand have increased beef prices, particularly in cull cows [1]. In the Canadian Grading System, cull cows are segregated as Canada D-grades based on a broad classification of carcass types [2]. In contrast to the top youthful grades (Canada Prime, AAA, AA, and A), where estimations for retail cut yield are routinely provided, Canada D-grades are lacking prediction of carcass yields before carcass breakdown. Because mature beef carcasses are often boned out for further processing, yield assessments of carcasses would be an important attribute to enhance fair compensation to the cattle producers. Furthermore, accurate estimations of carcass composition have been suggested to assure an efficient utilization of specific muscles from cull cow carcasses. In this sense, Roberts et al. [3] reported that, despite darker lean, many muscles from D-grade carcasses had higher intramuscular fat content than in the youthful A/AA carcasses. Given this retail performance of muscles from cull cow carcasses, opportunities may exist to better utilize specific muscles from these carcasses.

For decades, in North America, the carcass classification has been carried out by trained personnel (graders), thus implying a certain degree of subjectivity on the quantified parameters [4]. The latest improvements in technologies to estimate body/carcass composition have shown applicability on different species, genetics, production systems, etc. [5,6]. Computer vision systems (CVS) were implemented in the early 1980s as a computerized, non-destructive, non-invasive, objective, cost-effective, and automatable technology, based on image analysis that provides measurements of the beef carcass or rib-eye proportions [6]. The CVS have been recognized as useful tools to improve the grading accuracy, precision, and consistency, thus benefiting all segments of the beef production and consumption supply chain [7]. Typically, at least one of the two CVS approaches is used. Whole-side carcass image analysis, also known as hot carcass camera (HCC) system, which is designed to be integrated into the slaughter chain to work autonomously, and/or the rib-surface image analysis system, also known as cold carcass camera (CCC), which mimics the traditional visual assessment of the knife-ribbed surface of the rib-eye at the 12th thoracic vertebrae. The HCC uses a color camera and a lighting system, including structured (striped) light. The half carcass holds steady in front of a colored background and one or two images (if ambient light must be compensated) are taken to obtain 2D information, and a third image is taken with the structured light to capture 3D information of the carcass from the degree of curvature of the striped light [8,9]. Using proprietary software, the CCC provides an objective measure of rib-eye length, width, and area, and fat thickness, which are then used to predict carcass yield, as well as marbling, lean, and fat contents, and color assessments [6]. Currently, the CCC system is widely utilized by the beef industry in North America [8,9,10,11], particularly in youthful carcasses. However, unlike youthful beef, mature cull cows are generally marketed without knife-ribbing the carcass at the grade site. Hence, prediction of lean yield using rib-eye assessment or CCC is not achievable and development of alternative methods is particularly pertinent for the industry.

On the other hand, Dual-energy X-ray absorptiometry (DXA) technology is a promising indirect method to estimate carcass composition due to its relatively low cost, high reliability of data collection, and ease of use [5]. In the literature, the feasibility, accuracy, and precision of DXA technology has been reported on salmon [12], broiler chickens [13], sheep [14], swine [15], and cattle [16]. In addition, Soladoye et al. [15], Kipper et al. [17], and López-Campos et al. [18] assessed the accuracy of DXA technology on mass measurement of primal cuts from pigs and steers. Most of the published studies reported on the use of DXA in youthful populations, with information being scarce or almost lacking for mature animals, particularly in the case of cull cows. Contrary to the CVS, DXA technology is at the early stages of industry implementation.

Thus, the objective of the present study was to evaluate the potential of computer vision systems, namely the whole-side carcass camera compared to the rib-eye camera, as well as the emerging DXA technology to predict whole-carcass and primal composition (fat, lean, and bone) of mature cows. Furthermore, the combination of both computer vision systems was also explored in order to evaluate this approach as an alternative for the beef industry to further improve the prediction accuracy on primals and carcass composition of mature beef.

## 2. Materials and Methods

### 2.1. Animals

A total of 111 cull cow left carcass sides (hot carcass weight: HCW = 346 ± 33.3 kg), sourced from a commercial abattoir (*n* = 72) and from the AAFC-Lacombe Research and Development Centre (AAFC-Lacombe RDC) cow herd (*n* = 39), were used in the present study. AAFC-Lacombe RDC animals were cared for according to the Canadian Council on Animal Care Guidelines [19] (AAFC-Lacombe RDC study plan No. 201705).

### 2.2. Carcass Sides, Cut-Out, and CVS and DXA Scanning

Cull cows sampled from the AAFC-Lacombe RDC herd were slaughtered at the AAFC-Lacombe RDC federally inspected abattoir. Following slaughter, carcasses were dressed and split and HCW were recorded. In turn, commercial carcass sides, harvested following the Guidelines for the humane care and handling of food animals at slaughter (Canadian Food Inspection Agency, CFIA) [20], were shipped to AAFC-Lacombe RDC facilities in a refrigerated truck following guidelines for transportation of carcasses over thirty months of age (CFIA) [21]. At the time of slaughter, HCW were recorded by the personnel of the slaughter plant. In both sample populations, pictures of each carcass side were taken using a HCC unit VBS 2000, *e+v*^®^ Technology GmbH, Oranienburg, Germany.

Raw output data of the HCC images were composed of 187 variables describing carcass dimensions: angle (W00-W99), length (L00-L19), area (F00-F13, F20-F29), carcass contour and volumes (V00-V13, V20-V29), and color (Fe00-Fe18). Following 72 h of chilling at 2 °C, left carcass sides were weighed (CCW) to determine shrink loss. Physiological maturity of the carcasses were assessed based on the extent to which caps of the spinal processes and ribs had ossified (i.e., >50% ossification, a carcass receives a D grade) in accordance with López-Campos et al. [22] and the Canadian Beef Grading Agency (CBGA) [2]. Left carcass sides were then knife-ribbed between the 12th and 13th ribs. After 20 min of atmospheric oxygen exposure, full Canadian grading data were collected by a certified grader from the CBGA. The assessments included grade fat (minimum fat thickness over the rib in 4^th^ quadrant from the spinous process, mm), fat thickness (at the three-quarters position from the spinous process, mm), rib-eye area (REA; in cm^2^ of the *Longissimus thoracis*), and marbling score, subjectively assessed using United States Department of Agriculture (USDA) beef marbling pictorial standards as reference points [23]. Muscle scores (1–4) were also determined based on *L. thoracis* length and width, measured at the grade site [24,25]. Then, rib-eye pictures from each carcass side were taken using CCC: VBG 2000 (*e+v*^®^ Technology GmbH, Oranienburg, Germany). Each image was then processed by manufacturer software, in real-time, to produce raw output data composed of 99 variables describing a number of measurements related to measurements on the rib-eye (*n* = 22), fat thickness (*n* = 15), and muscle and fat color (*n* = 15), as well as other variables (*n* = 47, e.g., marbling assessment, back fat dressing corrections, etc.).

Estimated total lean meat yield (LMY) was calculated according to the Jones et al. [24,25] equation LMY (%) = 63.5 + 1.05 × (muscle score) − 0.76 × (grade fat). The retail cut yield (RCY) percentage was calculated using the equation RCY (%) = 51.34 − 5.78 × (fat thickness at the ¾, inches) − 0.46 × (kidney, pelvic, and heart fat percent, KPH, %) − 0.0093 × (hot carcass weight, HCW, pounds) + 0.74 × (REA, square inches) [25].

Left carcass sides were fabricated into primal cuts with carcass breakpoints identified following the Institutional Meat Purchase Specifications (IMPS) for Fresh Beef Products, Series 100 [26]. The primals collected from the left fabricated carcass side were the chuck (IMPS #113), rib (IMPS #103), brisket (IMPS #118), flank (IMPS #193, non-trimmed), foreshank (IMPS #117), loin (IMPS #172A), round (IMPS #158A), and plate (IMPS #121). Following procedures described by López-Campos et al. [18], each primal cut was scanned with a GE Lunar iDXA unit (GE Lunar, General Electric, Madison, WI, USA) using the whole-body scan option on standard mode to estimate fat, lean, and bone weights. After DXA scanning, all left primals were fully dissected into subcutaneous fat (SQ), intermuscular fat (IM), body cavity fat (BC), lean, and bone, then weighed by trained personnel. An adequate dissection processing was carried out by highly skilled meat cutters ensuring that the difference between primal weight and the sum of total bone, total lean, and total fat was not higher than a 2%.

### 2.3. Statistical Analyses

All the statistical analyses were performed using SAS v. 9.4 (SAS Institute Inc., Cary, NC, USA, 2014) [27]. Either CVS or DXA estimates of lean, fat, and bone weights from each primal cut, and the overall fat, lean, and bone weights were included as independent variables in a partial least square regression (PLSR) to generate prediction equations. Therefore, four different groups were defined depending on the regression estimating variables used: HCC, CCC, combination of HCC + CCC, and DXA. All models were used to predict the reference values from the manual dissections and the calculations of LMY and RCY equations.

All PLSR models were fit using an internal full leave-one-out cross-validation, to avoid overfitting in the calibration set, and the number of latent variables (LV) used to minimize predicted residual error sums of squares (PRESS) was reported for the calibrated PLSR models.

The predictive ability of the PLSR models was evaluated in terms of coefficient of determination (R^2^) and the mean square prediction error (MSPE), which was decomposed into error in central tendency (ECT), error due to regression (ER), and error due to disturbances (ED) [18]. These three fractions were calculated and expressed as percentages, as suggested by Benchaar et al. [28], as a means of describing the residual error in the models. ECT indicates how the average of CVS/DXA values deviates from the average of dissection values. ER measures the deviation of the least square regression coefficient from one, which is the value that it would have been if dissection and CVS/DXA measurements were in complete agreement. The ED is the variation in dissection measurements that is not accounted for by the least square regression of CVS/DXA measurements. In fact, this error is the unexplained variance and represents the portion of MSPE that cannot be eliminated by linear correction of the predictions [29]. Finally, when expressed as a percentage of the MSPE, the ECT, ER, and ED are called bias proportion, regression proportion (deviation of the regression slope from one), and disturbance proportion, respectively [30].

## 3. Results

### 3.1. Cow Carcass Population

All the carcasses used in the present study showed ossification processes at the caps of the thoracic vertebrae ranging from 50% to 100% ossified, resulting in carcasses graded as Canada D mature type grades [2]. Values of HCW (277.3–410.2 kg), CCW (271.3–401.9 kg), grade fat (0.0–29.0 mm), fat thickness (0.0–27.9 mm), REA (60–120 cm^2^), LMY (49.0–61.0%), RCY (42.9–54.5%), and marbling scores (100–733, USDA marbling score), of the carcass population (*n* = 111) used were within the actual range (Table 1) of the Canadian beef carcass market [1].

### 3.2. Primal Weight Estimation

Overall, CVS lean and fat predictions (Table 2) showed high R^2^ values for most of the primal cuts, while R^2^ values for bone were much lower. The HCC had similar performance to the CCC for fat predictions in the primal cuts, ranging from R^2^ = 0.47 to 0.88 compared to R^2^ = 0.51–0.92, respectively. More specifically, fat foreshank regression models showed the lowest R^2^ values, while plate, rib and round showed a 14.4% improvement with the HCC compared to CCC. Likewise, lean weight predictions in the primal cuts were superior using the HCC compared to the CCC, except for the rib with R^2^ ranging from a low of 0.53 in the foreshank to a high of 0.90 in the round for the HCC, and 0.32 in the foreshank to 0.69 in the rib for the CCC. Additionally, the HCC outperformed the CCC in the prediction of bone weight, showing R^2^ values as high as 0.79 in the round, while the highest R^2^ for the CCC bone weight was 0.38 in the chuck. Neither of the camera systems studied was able to accurately predict flank bone weight, and the CCC could not either accurately predict bone weight in the brisket, loin, rib, plate, or foreshank (R^2^ < 0.10, LV = 1 related variable was considered for prediction equation development).

When considering the error and/or variance partitioning, no remarkable differences were found for bone estimations. Nevertheless, CCC showed ECT values 97.4% higher and ED values 5.8% lower than HCC for fat primal estimations, although no difference was observed for MSPE. In the case of lean primal estimations, MSPE value for CCC resulted 63.2% higher than for HCC, although ED values for HCC resulted only 0.7% higher than CCC, and 81.7% of the difference was due to ER instead of ECT (24.4%) when compared to CCC (Table 2).

The combination of both CVS technologies did not improve fat estimations for most of the primals; only the round predictions (R^2^ = 0.88) showed some improvements compared to the HCC or CCC estimations; 3.4% and 18.2%, respectively. Conversely, lean estimations of brisket, chuck, plate, and rib showed, respectively, a 10.8%, 3.3%, 10.1%, and 16.2% higher R^2^ values for HCC + CCC than for HCC. Additionally, HCC + CCC improved bone estimations in the case of brisket (R^2^ = 0.42), chuck (R^2^ = 0.71), and loin (R^2^ = 0.76) compared to the individual CVS.

In the HCC+CCC, the contribution of ED to MSPE value was again much higher than the inputs coming from ER and ECT values (Table 2). For fat estimations, HCC + CCC showed lower ED and ER values and higher ECT values than HCC, and lower ECT and ER but higher ED values than CCC. In the case of lean estimations, HCC + CCC showed MSPE similar values to HCC, but these were lower than CCC. The ED values were lower than HCC and similar to CCC. The ER values were again higher than ECT, as observed in the HCC to CCC comparison. In addition, no remarkable differences were found for bone estimations. Interestingly, for fat estimations, the LV number for HCC + CCC was lower than for HCC or CCC.

In contrast, DXA primal estimations (Table 3), on average, had R^2^ values for fat (0.95), lean (0.97), and bone (0.82) higher than those for CVS, and even outperformed the prediction equations utilizing all camera variables (HCC + CCC; Table 2). Except for the foreshank fat weight (R^2^ = 0.74), DXA lean and fat weight predictions for the rest of the primals showed R^2^ values between 0.94 and 0.99 and 0.96 and 0.99, respectively. Similar to the CVS, lower values of R^2^ were observed for bone than for fat and/or lean variables; however, even flank bone weight (R^2^ = 0.31) was predicted more accurately using DXA than by using both camera systems combined. For the other primal bone weight predictions, DXA R^2^ ranged from 0.85 to 0.94, whereas the combined camera R^2^ values ranged from 0.36 to 0.76. Overall, there were improvements in most tissue primal predictions using DXA when compared to the camera systems. On average, for all the primals, there was an overall proportional improvement in DXA R^2^ values of 26.0%, 48.9%, and 24.8% compared to HCC, CCC, or HCC + CCC, respectively, as well as an increase in the DXA R^2^ values of 16.0%, 29.0%, and 54.7% for fat, lean, and bone estimations, respectively. The MSPE showed relatively low values and was defined by ED in a percentage higher than 98.7% for fat estimations, and higher than 99.8% for lean and bone tissue estimations (Table 3).

### 3.3. Overall Carcass Tissue Composition and Yield Estimations

Overall, relatively high R^2^ values (>0.75) were obtained between the estimations with the different technologies and the actual dissection values and yield equation estimates of LMY and RCY. Particularly, high relationships (R^2^ > 0.80) were observed between the estimations with DXA and HCC and the actual dissection values (Table 4). With the exception of LMY (R^2^ = 0.66 vs. 0.85), the HCC had similar or higher predictions for overall total carcass composition than the CCC (Table 4). In particular, the HCC predicted fat weights similar to (R^2^ = 0.92 vs. 0.93) and lean weights (R^2^ = 0.89 vs. 0.67) and bone weights (R^2^ = 0.82 vs. 0.31) better than the CCC camera. In fact, the HCC performed similar to DXA for all the total carcass composition estimates (R^2^ > 0.80), and only dropped in prediction accuracy for the LMY and RCY (R^2^ = 0.66 and 0.68 for the HCC and R^2^ = 0.81 and 0.86 for the DXA). Adding the CCC variables to the prediction (HCC + CCC) resulted in very similar prediction accuracies to those of DXA for all overall total carcass composition, including the estimates of LMY and RCY.

In general, DXA estimations showed lower MSPE values than any CVS (2.0% vs. 14.6% on average, respectively), and, particularly, higher MSPE values were observed for the CCC procedure than for the HCC or HCC + CCC ones (21.4%, 11.8%, and 10.5% on average, respectively). Besides, IM, total fat, and total lean estimates showed the highest MSPE values, whereas BC fat showed the lowest. Implicating MSPE components, 88% for ED input was observed for the estimation of total fat using CCC variables, while values higher than 90% were observed for the others.

## 4. Discussion

Canadian mature cow grades (D grades) are assigned to one of the D_1_, D_2_, D_3_, or D_4_ grades depending on variables such as muscling (excellent, medium, or deficient), fat color (white or yellow), and fat measure (lower than, equal to, or higher than 15 mm) [2]. In the present study, similar numbers of carcasses for each grade were considered (26.2%, 25.0%, 23.8%, and 25.0%, respectively for D_1_, D_2_, D_3_, and D_4_). The ranges of the HCW, CCW, grade fat and fat thickness, LMY and RCY, REA, and marbling scores of the research carcass population used in the present study were representative of those found in the Canadian beef market [1].

The technologies used in the present study provided estimation values of the total amount of tissue and an overall description of the composition of the whole carcass and primal cuts without requiring the destructive procedure of dissection.

In the literature, most of the studies considering the use of CVS in beef carcass classification have focused on the quantification of LMY, RCY, and/or the total amount of fat, lean, and bone using CCC systems. Among others, Farrow et al. [31], Lu and Tan [32], McEvers et al. [10], and Shackelford et al. [33] used several variables obtained from the analysis of rib-eye images to define different regression equations to improve the accuracy, precision, and robustness of total tissue amount, LMY, or RCY estimations. The results reported by these authors (R^2^ = 0.43–0.91) are within the range of those observed in the present study. All the authors agreed that CVS-related equations were an improvement on current prediction systems.

In agreement with the present study, Borggaard et al. [34] described similar R^2^ values for total fat and RCY (%) using a BCC-2 camera and a HCC system, but carried out the statistical analysis by means of principal component analysis (PCA) and neural networks. Likewise, Pabiou et al. [35] used the VBS 2000 carcass grading unit (HCC) to predict carcass cut yields in cattle. Diverging from our study, HCC and CCW variables were used in the estimation models, and it was stated that stepwise regression showed slightly better R^2^ values than the PLSR procedure, thus explaining 71%, 72%, and 75% of the variance for RCY (%), total fat (%), and total bone (%), respectively.

Vote et al. [36] compared BCSys (HCC) and CVS BeefCam (CCC) to study their potential as grading systems for Uruguayan beef carcasses. They reported higher RCY R^2^ values for CVS estimations than for values from the USDA equation (values coming from graders). In agreement with the present study, for total fat and bone estimations, higher R^2^ values were shown when using HCC or HCC + CCC technologies than when the CCC system was considered. In addition, in Vote et al. [36], bone amount estimations resulted in lower R^2^ values than fat amount estimations, and HCW was also included in the models.

RCY and LMY values are commonly obtained from equations in which rib-eye and fat thickness measurements are considered [25]. Because the equations are built from these variables, it is not surprising that the CCC predicts RCY and LMY better than HCC, as the linear measures of rib-eye and rib-eye area along with the fat thickness obtained with the CCC are likely improving these estimates. Nevertheless, in the case of cull cows, it is possible that the industry could be more interested in lean to fat ratios, in which case, HCC predictions outperformed CCC. Hence, cows could be graded accurately in terms of lean/fat ratio using a camera system that does not require knife-ribbing.

The literature regarding the estimation of cattle primal composition is scarce. Using a dual CVS system (HCC + CCC), Cannell et al. [37] tested a total of 296 carcasses: 158 steers (103 light (HCW ≤ 339 kg) and 55 heavy (HCW > 340 kg)) and 138 heifers (51 light and 87 heavy), and described, in agreement with our results, R^2^ > 0.65 for primal fabrication yields on average using a selection of HCC and CCC variables (higher coefficient of correlation), HCW, and stepwise regression (higher R^2^). Using a similar statistical approach, the HCC system, including the CCW variable (VBS 2000), Pabiou et al. [35] defined four cut-out groups according to their retail value (low, medium, high, and very high value) and obtained higher R^2^ values (0.84, 0.65, and 0.87) than in the present study for wholesale primal weights for steers, heifers, and bulls, respectively. In turn, Craigie et al. [11] used VBS 2000 technology (HCC system) and described R^2^ > 0.80 for the estimation of saleable (retail cut) sirloin weight, considering HCW in the regression models.

In other species, Rius-Vilarrasa et al. [38], using VSS 2000 (HCC system for lambs) and PLSR statistical analysis, reported R^2^ values of 0.86 for breast and 0.96 for leg primals. Lorenzo et al. [39] reported R^2^ values between 0.53 and 0.89 for the prediction of foal carcass composition and wholesale cut yields using HCC. Nevertheless, CCW was also considered as a describing variable in the prediction models, whereas HCW was used in the present study. The CCW has been described as a good estimator in the case of lamb carcasses [40]; however, its suitability has been questioned for cattle [35].

Kipper et al. [17] assessed the accuracy of the methodology using the concepts of trueness, defined in our case as the degree of agreement between the dissection and the instrumental estimation values, and precision, as indicative of the degree of internal agreement (dispersion). In addition, the trueness was considered to be the sum of ECT and ER; precision was associated with ED and overall accuracy was related to MSPE [17]. Paying attention to error parameters, higher ECT values in CCC and HCC + CCC than in HCC were detected in fat estimates, whereas the opposite behavior was observed for ED. Therefore, the similar values of R^2^ and high values of ED imply that the three instrumental approaches could be considered highly accurate and precise, the PLSR analysis being suitable for estimation. However, CCC and HCC + CCC fat estimations showed lower trueness than HCC estimations (Table 2).

In agreement with the present results, the feasibility of DXA technology in assessing carcass composition has been stated for broiler chickens [13], pigs [15], and sheep [14], and good R^2^ values have also been described for calves [16,18]. Aligning with our results, in all these studies, higher R^2^ values were described for total fat and total lean estimations than for total bone estimations.

López-Campos et al. [18] described similar results for the estimation of fat, lean, and bone mass of primals using DXA with youthful cattle. The basis of the DXA technology lies in the different absorption ratios from a low and a high energy X-ray beams when interacting with the tissues. The software estimates the mass of two different tissues at each scanned voxel; therefore, it is possible to differentiate between fat and lean when no bone is present but, where the sample matrix contains bone, fat, and lean, the mass fraction can only be established as bone and soft tissue, with the individual measurements of fat and lean obtained from other regions of the scan. Therefore, the higher the amount of bone detected, the more difficult the differentiation between fat and lean. In addition, a medical DXA unit has been used in the present study, thus being calibrated for the measurement of human bone mineral content and bone mineral density, but not for the whole bone content of livestock.

Again, the fact that ED explained more than 99% of the MSPE values would imply that the differences among R^2^ values were highly related to the dispersion (precision) and poorly related to the trueness. Therefore, the external factors such as calibration method, software analysis, the defining variables considered for the estimation models (HCW, CCW, marbling, color, gender, etc.), and the meat cutters’ decision making (tissue differentiation and cutting) would be the defining variables of dispersion. Accordingly, the low R^2^ value of flank bone might be a consequence of the low amount of bone included in this primal, thus implying a high variability in both DXA estimations and weight measurements. The foreshank showed the highest bone to soft tissue ratio, although the different contributions to lean or fat estimates remain unclear: higher and lower accuracy of lean and fat estimates of foreshank, respectively.

Regarding bone prediction, similar results to those from this study were described in pigs [41] and chickens [42]. Kipper et al. [41] and Schallier et al. [42] described better R^2^ values when the predicted amount of bone was correlated with ash content. This implies that the presence of small pieces of lean or fat that were adhered to the bone decreases the accuracy and precision of the analysis, whereas it increases the error between actual and predicted values.

Similarly to DXA, computed tomography (CT) is a technology also based on X-ray attenuation. Prieto et al. [43] described lower R^2^ values for IM and total fat than for SQ and total lean predictions (0.77, 0.89, 0.94, and 0.99, respectively) using spiral CT. Concurring with the present study, Navajas et al. [44] described lower R^2^ values for carcass total bone than for fat and/or lean estimates when using CT technology (R^2^ = 077, 0.92, and 0.96). In addition, Navajas et al. [45] described R^2^ values of 0.92, 0.99, and 0.97, respectively, for fat, lean, and bone for the primal estimations.

To date, DXA has been limited by practical constraints for deployment in the industry (horizontal table scans, operation at room temperature, and rate of scan in minutes rather than seconds). However, Scott Technologies Ltd. (New Zealand) has developed an upright DXA scanner, capable of scanning at a rate of 540 lamb carcasses per hour while maintaining performance accuracy. This technology adaptation was originally used to mark anatomical features to program robotic cutting. The technology is now being envisioned as a means of lean yield prediction in beef and lamb plants in Australia and New Zealand.

May et al. [46] reported that estimated yield differences could be attributed partially to differences in seam fat deposition (different fat deposition along the carcass). Likewise, in practice, the fabrication of the boneless, closely trimmed round, loin, rib, and chuck retail cuts is performed manually by meat cutters, thus implying another subjective source of variability. Although these factors might introduce variations in the cutability, the present results suggest that both CVS and DXA technologies have the potential to estimate beef carcass traits such as total or retail cut yield performance.

Finally, it is worth mentioning that, based on its performance, DXA might be seen as the gold standard candidate technology for carcass composition estimation. Currently, DXA technology is still under development and it is also being used as a means of envisioning bone location for robotic carcass fabrication. The costs and other operational factors are limiting its industrial implementation. However, if a facility had the capabilities to set up both camera systems, and knife rib cows at the 12/13th, combining the HCC and CCC data, could result in prediction accuracies very similar to DXA. This approach would be of benefit to the plants in determining which carcasses would be profitable for specific fabrication lines.

## 5. Conclusions

The results of the present study suggest that tissue composition (fat, lean, and bone), either from primal cuts or full carcass sides, and yield percentages of LMY and RCY of mature cows can be accurately predicted by CVS or DXA technologies by applying partial least square regression statistical analysis.

Although DXA results showed higher accuracy, precision, and robustness than results from CVS technologies, DXA technology is still in development for cattle and would require further design adjustments for full implementation and integration into commercial slaughter plants with moving carcass lines.

On the contrary, CVS technologies (HCC and CCC cameras) are widely implemented in North America. In the present study, predictions using HCC variables led to similar or even better results (higher R^2^ and lower MSPE values) than those from CCC. The implementation of HCC technology for the carcass composition estimations of mature cows has the benefit that knife ribbing of the carcasses is not required, not even for RCY (%) or LMY (%) estimations. Furthermore, the combination of both CVS technologies led to significant improvements in the tissue predictions of primal cuts and total carcass composition, particularly for lean/fat ratios, suggesting this approach as an alternative for the enhancement of the prediction accuracy on primals and carcass composition of cull cows.

## Figures and Tables

**Table 1:** Descriptive statistics of carcass characteristics of the population used to obtain the prediction equations between the camera vision system values and whole carcass and primal composition (fat, lean, and bone).

	Mean (*n* = 111)	SD ^1^	Min	Max
HCW ^2^ (kg)	345.8	33.3	277.3	410.2
CCW ^3^ (kg)	338.7	30.0	271.3	401.9
Grade fat (mm)	9.6	8.06	0.0	29.0
Fat thickness (mm)	10.2	5.59	0.0	27.9
Rib-eye width ^4^	1.8	0.78	1	3
Rib-eye length ^4^	2.7	0.53	1	3
Muscle score ^4^	2.4	0.99	1	4
Ribeye area (cm^2^)	83.6	11.2	60.0	120.0
LMY ^5^ (%)	56.3	5.75	49.0	61.0
RCY ^6^ (%)	49.6	2.29	42.9	54.5
Marbling scores ^7^	455.6	143.2	100.0	733.0
Ossification (%) ^8^	92.8	13.4	50.0	100.0

^1^ SD: standard deviation; ^2^ HCW: Hot carcass weight; ^3^ CCW: Cold carcass weight; ^4^ Rib-eye width and length, and muscle score in agreement with Jones [24] and Segura et al. [25]; ^5^ LMY: estimated total lean meat yield [25]; ^6^ RCY: retail cut yield [25]; ^7^ Marbling scores: Official United States Standards for Grades of Beef Carcasses (marbling scores: 0 = Devoid, 100 = Practically Devoid, 200 = Traces, 300 = Slight, 400 = Small, 500 = Modest, 600 = Moderate, 700 = Slightly Abundant, 800 = Moderately Abundant, 900 = Abundant) [23]; ^8^ Ossification (%): Ossification processes of the carcasses assessed on the caps of the spinal processes and ribs (i.e., >50% ossification, a carcass receives a D grade) according to López-Campos et al. [22] and the Canadian beef Grading Agency [2].

**Table 2:** Partial least square regression models estimating lean, fat, and bone for individual primal cuts from computer vision system (CVS) values. Coefficient of determination (R^2^), mean square prediction error (MSPE), error in central tendency (ECT), error due to regression (ER), error due to disturbances (ED), and the number of latent variables (LV) are presented for each model.

Tissue	Primal ^4^	HCC ^1^ (*n* = 105)						CCC ^2^ (*n* = 102)						HCC + CCC ^3^ (*n* = 95)					
		R^2^	MSPE	ECT (%)	ER (%)	ED (%)	LV	R^2^	MSPE	ECT (%)	ER (%)	ED (%)	LV	R^2^	MSPE	ECT (%)	ER (%)	ED (%)	LV
Fat (kg)	Brisket	0.86	0.1942	0.35	0.14	99.51	8	0.88	0.1817	6.46	0.09	93.44	10	0.80	0.2872	2.68	0.01	97.31	2
	Chuck	0.88	2.3678	0.25	0.14	99.61	8	0.91	2.1435	9.98	0.29	89.73	10	0.87	2.8515	5.95	0.01	94.04	3
	Flank	0.86	0.9713	0.15	0.18	99.67	7	0.92	0.6498	9.23	0.12	90.66	10	0.88	0.8844	5.50	0.05	94.45	3
	Loin	0.81	2.5542	0.00	0.18	99.82	9	0.91	1.3322	8.29	0.23	91.48	10	0.85	2.1950	5.90	0.02	94.08	3
	Plate	0.87	0.6728	0.00	0.10	99.90	10	0.73	1.4707	2.74	0.02	97.24	4	0.84	0.9188	5.35	0.01	94.64	3
	Rib	0.87	1.1126	0.07	0.15	99.78	10	0.78	2.0276	3.66	0.04	96.30	3	0.86	1.3099	5.21	0.02	94.77	3
	Round	0.85	0.6854	0.27	0.01	99.71	4	0.72	1.3811	4.01	0.26	95.73	2	0.88	0.6259	6.71	0.49	92.80	2
	Foreshank	0.47	0.0332	0.08	0.00	99.92	2	0.51	0.0309	0.51	0.00	99.49	4	0.50	0.0316	1.17	0.00	98.83	2
Lean (kg)	Brisket	0.67	0.2783	0.06	0.10	99.85	2	0.62	0.3286	0.55	0.76	98.69	4	0.76	0.2107	0.48	0.70	98.83	3
	Chuck	0.85	4.6386	1.10	0.04	98.86	4	0.52	14.710	1.02	0.83	98.15	3	0.88	3.9094	0.70	4.53	94.77	5
	Flank	0.82	0.3376	2.26	0.03	97.71	9	0.55	0.8112	1.57	0.37	98.06	2	0.74	0.4638	0.13	1.11	98.75	3
	Loin	0.82	1.5263	0.41	0.16	99.43	5	0.58	3.5920	0.72	0.22	99.06	3	0.82	1.5196	0.47	0.19	99.34	4
	Plate	0.75	0.5167	0.04	0.08	99.87	3	0.46	1.1186	0.32	0.25	99.43	4	0.83	0.3492	0.66	0.73	98.61	5
	Rib	0.66	1.2365	0.41	0.07	99.52	2	0.69	1.1224	0.45	1.02	98.53	3	0.79	0.7751	0.00	0.86	99.14	3
	Round	0.90	2.0669	0.59	0.26	99.15	10	0.65	7.2982	1.20	0.58	98.23	4	0.86	2.9706	1.28	0.90	97.82	4
	Foreshank	0.53	0.1596	0.14	0.03	99.83	2	0.32	0.2328	0.80	0.17	99.03	2	0.51	0.1681	0.53	0.20	99.27	2
Bone (kg)	Brisket	0.37	0.0566	0.05	0.00	99.95	2	0.01 ^5^	0.0855	0.30	0.00	99.70	1	0.42	0.0526	0.25	0.00	99.75	2
	Chuck	0.68	0.4167	0.01	0.01	99.98	4	0.38	0.8187	0.14	0.08	99.78	4	0.71	0.3886	0.46	0.24	99.31	3
	Flank	0.09 ^5^	0.0086	0.03	0.01	99.97	1	0.03 ^5^	0.0091	0.00	0.01	99.99	1	0.09 ^5^	0.0086	0.06	0.05	99.89	1
	Loin	0.64	0.1272	0.05	0.00	99.95	4	0.03 ^5^	0.3185	0.01	0.01	99.99	1	0.76	0.0848	0.03	0.25	99.72	6
	Plate	0.62	0.0598	0.02	0.01	99.97	2	0.09 ^5^	0.1329	0.01	0.01	99.99	1	0.62	0.0595	0.09	0.02	99.89	2
	Rib	0.36	0.1358	0.14	0.01	99.85	2	0.04 ^5^	0.1896	0.08	0.04	99.88	1	0.36	0.1369	0.59	0.33	99.08	2
	Round	0.79	0.2256	0.17	0.12	99.71	5	0.36	0.6723	0.05	0.07	99.88	4	0.75	0.2622	0.64	0.10	99.26	3
	Foreshank	0.60	0.0504	0.00	0.05	99.94	2	0.02 ^5^	0.1156	0.13	0.04	99.83	1	0.55	0.0574	0.28	0.32	99.40	2

^1^ HCC = hot carcass (whole-side) camera: regression models obtained using HCC variables. ^2^ CCC = cold carcass (rib-surface) camera: regression models obtained using CCC variables. ^3^ HCC + CCC = regression models obtained using the variables from both CVS. ^4^ Primals according to Institutional Meat Purchase Specifications (IMPS) for Fresh Beef Products, Series 100 [26]. ^5^ No statistically significant regression model (*p* > 0.05) was obtained. LV = 1 was considered to establish a prediction equation.

**Table 3:** Partial least square regression models estimating fat, lean, and bone for individual primal cuts from dual-energy X-ray absorptiometry (DXA) values (n = 111). Coefficient of determination (R^2^), mean square prediction error (MSPE), error in central tendency (ECT), error due to regression (ER), error due to disturbances (ED), and the number of latent variables (LV) are presented for each model.

Tissue	Primal ^1^	R^2^	MSPE	ECT (%)	ER (%)	ED (%)	LV
Fat (kg)	Brisket	0.99	0.0143	0.521	0.021	99.46	10
	Chuck	0.99	0.3074	0.335	0.019	99.65	10
	Flank	0.98	0.1540	0.097	0.049	99.85	6
	Loin	0.98	0.2395	0.219	0.032	99.75	10
	Plate	0.98	0.1039	1.054	0.254	98.69	10
	Rib	0.98	0.1384	0.940	0.095	98.96	10
	Round	0.96	0.1734	0.253	0.001	99.75	10
	Foreshank	0.74	0.0160	0.096	0.025	99.88	4
Lean (kg)	Brisket	0.99	0.0128	0.088	0.094	99.82	10
	Chuck	0.99	0.4146	0.023	0.135	99.84	10
	Flank	0.97	0.0519	0.004	0.066	99.93	10
	Loin	0.95	0.3825	0.003	0.042	99.96	6
	Plate	0.95	0.0964	0.041	0.024	99.93	7
	Rib	0.98	0.0569	0.006	0.126	99.87	10
	Round	0.99	0.2775	0.123	0.093	99.78	10
	Foreshank	0.94	0.0205	0.047	0.013	99.94	9
Bone (kg)	Brisket	0.89	0.0096	0.141	0.013	99.85	5
	Chuck	0.92	0.1081	0.009	0.019	99.97	8
	Flank	0.31	0.0066	0.043	0.007	99.95	3
	Loin	0.88	0.0420	0.106	0.005	99.89	9
	Plate	0.94	0.0088	0.044	0.017	99.94	9
	Rib	0.85	0.0313	0.006	0.009	99.98	5
	Round	0.92	0.0875	0.038	0.008	99.95	6
	Foreshank	0.86	0.0179	0.026	0.004	99.97	4

^1^ Primals according to Institutional Meat Purchase Specifications (IMPS) for Fresh Beef Products, Series 100 [26].

**Table 4:** Partial least square regression models estimating total fat, lean, and bone amounts, and total subcutaneous (SQ), body cavity (BC), and intermuscular (IM) fat amounts for whole carcass sides and total lean meat yield (LMY) and retail cut yield (RCY) from dual-energy X-ray absorptiometry (DXA) and computer vision system (CVS) values. Coefficient of determination (R^2^), mean square prediction error (MSPE), error in central tendency (ECT), error due to regression (ER), error due to disturbances (ED), and the number of latent variables (LV) are presented for each model.

	HCC ^1^ (*n* = 105)						CCC ^2^ (*n* = 102)						HCC + CCC ^3^ (*n* = 95)						DXA (*n* = 111)					
	R^2^	MSPE	ECT (%)	ER (%)	ED (%)	LV	R^2^	MSPE	ECT (%)	ER (%)	ED (%)	LV	R^2^	MSPE	ECT (%)	ER (%)	ED (%)	LV	R^2^	MSPE	ECT (%)	ER (%)	ED (%)	LV
Fat (kg)	0.92	29.407	0.130	0.249	99.62	10	0.93	30.104	11.66	0.427	87.91	10	0.91	35.532	9.073	0.011	90.92	3	0.99	2.5943	1.107	0.000	98.89	7
Lean (kg)	0.89	36.092	1.066	0.165	98.77	5	0.67	104.53	1.305	1.276	97.42	4	0.93	23.044	1.403	5.401	93.20	6	0.99	3.1380	0.046	0.180	99.77	8
Bone (kg)	0.82	2.4731	0.000	0.039	99.96	5	0.31	9.2266	0.061	0.151	99.79	1	0.84	2.1539	0.323	1.360	98.32	5	0.92	1.0459	0.029	0.013	99.96	5
SQ (kg)	0.88	6.5924	0.219	0.086	99.69	8	0.82	9.7306	4.224	0.007	95.77	3	0.88	6.5623	4.704	0.063	95.23	3	0.95	2.5014	0.038	0.025	99.94	10
BC (kg)	0.81	0.5213	0.404	0.136	99.46	10	0.75	0.6954	1.960	0.307	97.73	7	0.75	0.6965	4.453	0.084	95.46	4	0.81	0.5184	0.111	0.024	99.87	5
IM (kg)	0.91	10.189	0.145	0.269	99.59	10	0.91	12.097	10.30	0.462	89.24	10	0.90	12.709	8.903	0.026	91.07	3	0.98	1.7734	0.742	0.022	99.24	7
LMY (%)	0.66	7.3418	3.603	0.034	96.36	5	0.85	3.1867	4.719	0.113	95.17	5	0.90	2.2255	8.180	0.069	91.75	6	0.81	3.9807	0.176	0.482	99.34	5
RCY (%)	0.68	1.7008	0.641	0.001	99.36	10	0.65	1.8364	1.321	0.054	98.63	4	0.86	0.7776	6.983	0.589	92.43	6	0.86	0.7566	0.027	0.003	99.97	6

^1^ HCC = hot carcass (whole-side) camera: regression models obtained using HCC variables. ^2^ CCC = cold carcass (rib-surface) camera: regression models obtained using CCC variables. ^3^ HCC + CCC = regression models obtained using the variables from both CVS systems.
